# Performance of Hop Cultivars Grown with Artificial Lighting under Subtropical Conditions

**DOI:** 10.3390/plants12101971

**Published:** 2023-05-12

**Authors:** Nathalia Rodrigues Leles, Alessandro Jefferson Sato, Leo Rufato, Jessiane Mary Jastrombek, Viviani Vieira Marques, Robson Fernando Missio, Nelson Luis Mello Fernandes, Sergio Ruffo Roberto

**Affiliations:** 1 Agricultural Research Center, Agronomy Department, State University of Londrina, Celso Garcia Cid Road, km 380, Londrina 86057-970, Brazil; nathalia.leles@uel.br (N.R.L.); marquesvivivima@gmail.com (V.V.M.); 2 Agricultural Sciences Unit, Agronomical Sciences Department, Federal University of Parana, Pioneiro Street 2153, Palotina 86057-970, Brazil; asato@ufpr.br (A.J.S.); rfmissio@ufpr.br (R.F.M.); 3 AgroVeterinarian Center, Agronomy Department, Santa Catarina State University, Luiz de Camões Ave., 2090, Lages 88520-000, Brazil; leo.rufato@udesc.br (L.R.); jessiane.mj@edu.udesc.br (J.M.J.); 4 Agricultural Sciences Unit, Veterinary Sciences Department, Federal University of Parana, Pioneiro Street 2153, Palotina 86057-970, Brazil; nelson@ufpr.br

**Keywords:** *Humulus lupulus* L., beer, lupulin, alpha-acids, essential oils

## Abstract

The objective of this study was to determine the duration of the main phenological stages, plant growth development, yield, and cone quality of hop cultivars grown under artificial light (17 h per day) during vegetative development (early season) in a subtropical climate region. The study was conducted in Palotina, Paraná, Brazil (24° S), during the 2021 and 2022 growing seasons. The plants were cultivated in a 5.5 m high trellis system with artificial light supplementation during vegetative development. The hop cultivars Hallertau Mittelfrüher, Mapuche, Northern Brewer, Spalter, and Yakima Gold were used in the treatments. The duration of the phenological stages, vegetative growth (plant height, fresh mass of the plants, number of lateral branches per plant), components of productive yield (number of cones per side branch, number of cones per plant, fresh mass, length, and width of the cone, production of fresh cones per plant, and yield), and chemical components of the cones (alpha- and beta-acid contents, and essential oil concentrations) were recorded. The duration of the phenological stages was visually evaluated, and plant growth was analyzed using non-linear log-logistic regression. The remaining data were subjected to analysis of variance and the means were compared using Tukey’s test. The data were also subjected to multivariate analysis using the principal components test, correlation analysis, and hierarchical grouping. The cultivar Mapuche was considered an early hop in both seasons, and the cultivars Spalter and Yakima Gold were considered early cultivars in the second season. In both seasons, the productive yield components were positively correlated with the precocity of the cultivars, in which Mapuche in the 2021 season and Mapuche, Spalter, and Yakima Gold in the 2022 season had the highest mean of the number of cones per side branch and per plant, production per plant, and productivity. The cultivar Yakima Gold had a positive correlation with the chemical quality of cones, alpha and beta acid contents, and essential oil concentrations, for both seasons.

## 1. Introduction

Hops (*Humulus lupulus* L.) are perennial plants belonging to the Cannabaceae family [1]. Female flowers, called cones, have glandular trichomes that store and secrete lupulin, and consist of resins and essential oils that can be used for industrial and medicinal purposes [2]. In the brewing industry, these constituents are used to impart bitterness, flavor, and aroma to beers, and serve as cofactors in several product stabilization processes, making hops an essential ingredient for beverage production [3].

Hops are short-day plants with a critical photoperiod of 15 h; flowering is stimulated when the day length is <15 h [4]. Under favorable photoperiod conditions, bines can reach a peak growth rate of up to 25 cm per day [5]; however, photoperiod conditions are insufficient for vegetative growth in the early season, resulting in an insufficient number of nodes, limiting the ability of the plant to flower, and reducing cone production [1,4].

The duration of the photoperiod varies depending on latitude; therefore, the ideal latitudinal range to meet the demand for hops is from 35° to 55° N or S of the Equator [5,6,7], which coincides with the latitude of the main global hop producers, the United States and Germany, wherein 70% of the global cultivated hop area is located [4]. However, there have been successful cultivation initiatives in areas with a subtropical climate in the subtropics (latitude 23.5° and 35° N or S), such as West Central Florida (27° N) [1], using artificial lighting to ensure that hop plants achieve adequate vegetative growth and avoid early flowering [8].

In Brazil, several failed attempts were made to grow hops a few decades ago, and one of the limiting factors was a day length of less than 14 h. Consequently, despite being the third-largest beer producer in the world, with an estimated production of 15.3 billion L. year^−1^ [9,10], Brazil currently imports almost all of the raw material used in the industry (3243 tons) from the United States and Germany [11]. Additionally, the increase in the number of craft breweries that require this raw material has contributed to increasing interest in its cultivation in the country. Therefore, the development of studies that can help expand the Brazilian production chain has been encouraged, with the aim of adapting cultivation technologies and international cultivars to local conditions [12]. In particular, artificial lighting with LED lamps has been used to inhibit early flowering of plants in the field [4].

Understanding the agronomic behavior of different cultivars under local climatic conditions is a prerequisite for the successful development of hop cultivation in non-traditional areas. Each hop cultivar has a different concentration of resins and essential oils [13] and thus a different quality profile, which in turn is influenced by the interaction between the genotype and environment. In other words, a particular cultivar may exhibit different chemical composition when grown in different regions, which in turn may affect its bitterness and aromatic flavor when used in the brewing industry [14].

As hop cultivation in subtropical areas is very recent, reliable scientific information on the adaptation of cultivars to artificial lighting is limited. This study aimed to characterize the duration of the main phenological stages, plant growth performance, productive yield, and cone quality of hop cultivars grown under artificial lighting conditions in a subtropical region.

## 2. Results and Discussion

### 2.1. Hop Cultivar Phenology

#### 2.1.1. Season of 2021

**Figure 1:**
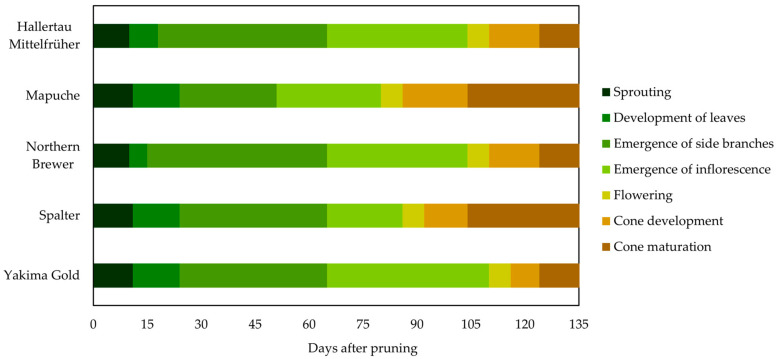
Duration in days of the main phenological stages of hop plant cultivars in the 2021 season.

The duration of the main phenological phases differed between hop cultivars during the 2021 season (Figure 1). Although all cultivars began sprouting at roughly the same time, it was observed that Mapuche and Spalter reached the stages of inflorescence emergence and flowering earlier, approximately 85 and 90 days from the beginning of vegetative development, respectively. In the other cultivars, inflorescences emerged approximately 110 days after the beginning of vegetative development.

Despite differences in phenological stages, the duration of the total cycle of the evaluated cultivars was similar; for all cultivars, the cones were harvested approximately 135 days after pruning. However, in the first season, the cones were harvested during the same period as they rapidly entered the senescence phase.

The phenological behavior of hop plants is influenced by the interaction between the genotype and environment, and this interaction manifests as the heterogeneity of genotypes under different climatic cultivation conditions [15]. Day length is a critical environmental factor that influences the duration of the phenological phases [1], and our results showed that the phenological behavior of the cultivars was similar to that of plants cultivated in some temperate climate regions (42° N) with a photoperiod longer than 15–16 h [16], and differed from that of plants found in subtropical climate regions (27° N) with a photoperiod shorter than 14 h [1]. This indicates that artificial lighting may have influenced the phenological cycle of the cultivars.

In regions with a photoperiod longer than 15–16 h, hop flowering occurs quickly and is synchronized approximately 90 days after sprouting, with an average duration of 14 days. Furthermore, the stages of cone development and maturation occur neatly, and the cones of a plant mature at virtually the same time [16]. In contrast, in regions with a photoperiod of less than 14 h, flowering is generally induced earlier, 26 days after sprouting, and this phase is three times longer than the typical flowering period in a temperate climate (42° N). Under these conditions, distinct phenological stages occur simultaneously, such as the formation of side shoots, development of new flowers, and senescence of cones, which can be attributed to inadequate day length in a subtropical climate (27° N) [1].

Studies on the use of artificial lighting to control hop flowering are limited. However, several studies on the use of artificial lighting in the protected cultivation of medicinal cannabis (*Cannabis sativa*: Cannabacea), another short-day flowering plant that requires a photoperiod of over 16 h in the early season, indicate that, as the photoperiod increases, the response to flowering is delayed, and the early season is extended by increasing vegetative biomass [17,18,19,20,21]. This corroborates the phenological behavior observed for hop cultivars evaluated under an extended photoperiod in this study.

#### 2.1.2. Season of 2022

**Figure 2:**
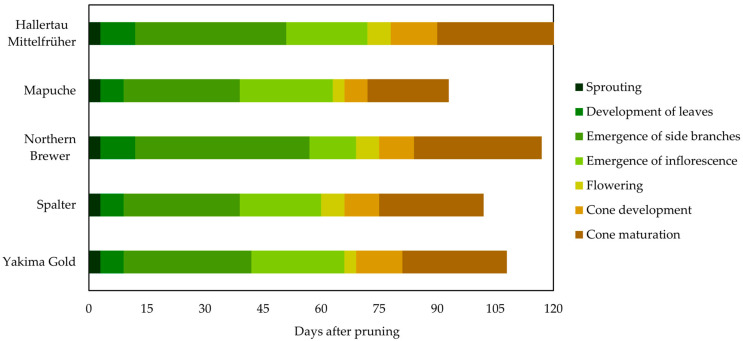
Duration in days of the main phenological stages of hop plant cultivars in the 2022 season.

In general, in the 2022 season, the cycles of all cultivars differed from those observed in the first season (Figure 2). Inflorescence emergence and flowering in Mapuche, Spalter, and Yakima Gold occurred earlier, approximately 60 and 65 d after the beginning of vegetative development, respectively, resulting in anticipation of cone maturation, which occurred in approximately 100 days. The period in which inflorescence emergence occurred was similar for the other cultivars, approximately 70 days after the beginning of vegetative development of the plants, totaling cycles of approximately 120 days.

In the 2022 season, we observed that the phenological behavior of the cultivars was similar to that of those cultivated in regions with a photoperiod longer than 15–16 h, indicating the need for artificial lighting in the early season for hop cultivars.

The shortening of the cycle of the cultivars in the first season may be related to the time when pruning was performed. In the second season, the cycle of the plants began in October, and a second pruning was necessary because of the low initial development of the plants caused by the low temperatures and high rainfall that occurred after the first pruning conducted at the end of July. Thus, the development of plants in the 2022 season occurred under conditions of higher temperatures, which resulted in an earlier onset of the phenological stages of hops [22,23]. Additionally, the influence of climate on the development of some cultivars was also observed, such as Yakima Gold, which had a late phenological behavior in the first season and an early behavior in the second season. Each cultivar requires a specific set of conditions for its development, mainly with regard to air temperature [22,23], suggesting that Yakima Gold optimizes its development when pruned at later times when the air temperature is higher.

### 2.2. Hop Growth Development

#### 2.2.1. Season of 2021

**Figure 3:**
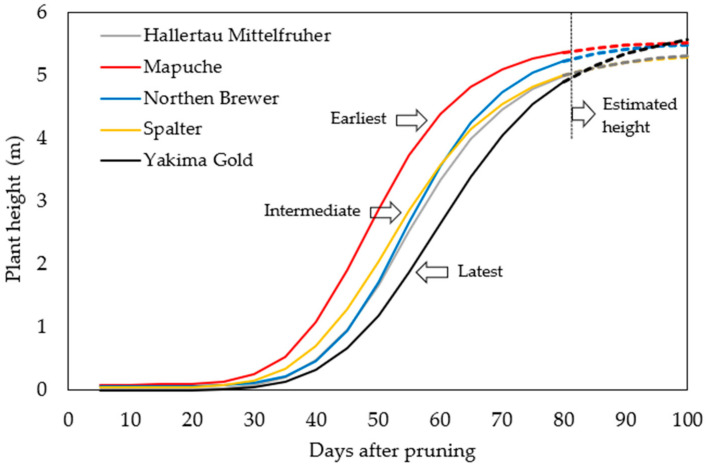
Hop growth development during the 2021 season.

Differences in plant growth and development were observed among cultivars in terms of the time required to reach the top of the trellis (Figure S1). Thirty days after the beginning of vegetative growth, Mapuche showed a more accelerated growth peak, indicating that this cultivar was the earliest in this season, while Hallertau Mittelfrüher, Northern Brewer, and Spalter were intermediate, and Yakima Gold was the latest (Figure 3).

Hop plant development is also influenced by photoperiod and timing of floral induction [2]. Before flowering, a photoperiod of approximately 17 h was used to facilitate adequate plant growth until the top of the trellis was reached. An insufficient photoperiod in the early season results in an insufficient number of nodes being formed, which affects the productive yield of the plants [1,4] because the inflorescences originate from the buds formed in each growth node.

The length of the photoperiod under artificial lighting with halogen lamps (Osram 2900 K 42 W) also influences stem elongation in the initial stages of growth in medicinal cannabis, allowing it to reach maximum plant height. Early floral initiation is induced in this species by providing a photoperiod shorter than the critical photoperiod, restricting the development and growth of internodes in the primary axis [20].

A significant difference in the number of side branches, but not in the fresh mass of the plants, was found between cultivars during the 2021 season (Table 1). The Mapuche and Northern Brewer cultivars had the highest means for the number of side branches per plant, and Spalter and Yakima Gold cultivars had the lowest values. Cultivars that produce the highest number of side branches per plant generally show the best cone production performance [24] because the number of cones formed is proportional to the number of fruitful side branches and their lengths [25].

#### 2.2.2. Season of 2022

**Figure 4:**
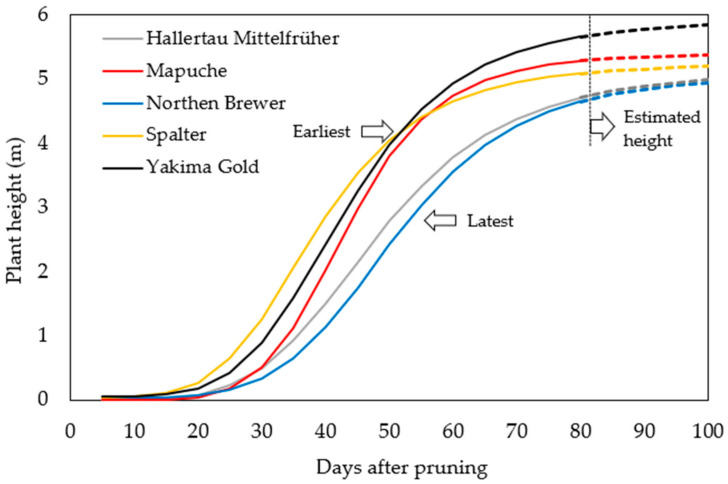
Hop growth development during the 2022 season.

In 2022, differences were observed between cultivars in terms of the time required to reach the top of the trellis (Figure S2). Twenty days after vegetative growth began, Mapuche, Spalter, and Yakima Gold grew faster, and were considered early cultivars, unlike Hallertau Mittelfrüher and Northern Brewer, which showed late growth and did not reach the top of the trellis until flowering (Figure 4).

The slower development of Hallertau Mittelfrüher and Northern Brewer in this season may be associated with the low accumulation of nutritional reserves in the rhizome due to the second cutback in October. Nutritional reserves are transferred to the rhizome at the end of the crop cycle during the plant dormancy period. Therefore, the development of these plants may have been impaired as they did not go through this accumulation period and possibly used a large portion of their nutritional reserves for vegetative development after the first pruning [7,26]. During this season, Yakima Gold and Spalter are considered early cultivars, similar to Mapuche, and climatic conditions during the pruning season, especially air temperature, may have influenced plant growth. In general, an increase in temperature stimulates hop plants to start growing after pruning, with the ideal temperature range being between 13.2 and 20.5 °C [1]. These observations indicate that some cultivars, such as Mapuche, can be pruned earlier, and others, such as Spalter and Yakima Gold, later, enabling a better distribution of agricultural practices that demand high labor, such as the selection of bines and harvest.

In addition to the influence of the environment, each cultivar has an ideal number of days for plants to grow until flowering [27,28]. The results of this study indicate that Mapuche, Spalter, and Yakima Gold require fewer days for vegetative development, and consequently, less exposure to the extended photoperiod.

Regarding the fresh mass of the plants, it was observed that the Mapuche, Spalter, and Yakima Gold cultivars, consistent with the growth and development of the plants, had the highest means, whereas the Hallertau Mittelfrüher and Northern Brewer cultivars exhibited the lowest mean values. Regarding the number of side branches, Spalter had the highest mean and Hallertau Mittelfrüher the lowest (Table 2). The cultivars with the highest fresh plant mass also had the highest number of side branches per plant, except for Mapuche.

In both seasons, hop cultivars flowered only after artificial lighting was suspended, based on the total height of the earliest cultivars. Additionally, plant growth ceased after the suspension of artificial lighting. Therefore, the artificial lighting used in both seasons, even with LED lamps of different spectra, can be considered necessary for plant vegetative development, resulting in the formation of a sufficient number of nodes for cone production. However, the cultivation of hops under artificial lighting in Brazil should be further investigated to determine which lamps have the most efficient spectra to control plant flowering, density, and distribution in the production area, for each crop. 

### 2.3. Yield Components

#### 2.3.1. Season of 2021

Significant differences in the number of cones per side branch, number of cones per plant, and cone mass were observed among the hop cultivars assessed during the 2021 season (Table 3). Regarding the number of cones per side branch and number of cones per plant, Mapuche had the highest means among the cultivars. Although lower than that of Mapuche, the means observed for Spalter were higher than those of the other cultivars. Hallertau Mittelfrüher and Mapuche showed higher cone mass than Yakima Gold.

The number of cones per side branch and per plant and cone mass constitute the main productive components of hops [29]; therefore, cultivars that stand out in these characteristics usually achieve the best productive yield.

When cultivated in tropical conditions (21° S) and without artificial lighting, the Mapuche and Yakima Gold cultivars showed an average of 49.7 and 60.3 cones per plant, respectively [30], which is considerably lower than that observed in the first season of cultivation in this study, and no comparative data were found under these conditions for the other cultivars under study. The number of cones per side branch and per plant has a positive relationship with vegetative development [29,30,31]; therefore, when environmental conditions do not favor plant growth, the number of cones is low. In the cultivation of medicinal cannabis plants under artificial lighting, the biomass at the time of changing the photoperiod from long to short days affects the number of flowers produced [21], corroborating the results of this study, in which the genotype of hops that stood out as late and vigorous also had the highest number of cones per side branch and per plant.

Cone mass is positively correlated with the physical attributes of the cone that determine its size, such as length and width [29]. Regarding the physical characteristics of the cones, only cone width was found to differ among the hop cultivars (Table 4). Hallertau Mittelfrüher, consistent with the cone mass, had the highest mean cone mass, and Spalter showed the lowest mean cone mass.

The size of the cone, defined by its length and width, directly influences its mass and, consequently, the productivity of the plants [25]. These characteristics are intrinsic to each cultivar [30]; however, cone width was lower in Spalter compared to the other cultivars, indicating that cone size did not influence productivity during the 2021 season. For hops, the qualitative factors of the cones are more important than the quantitative factors, considering that the end product directly depends on the alpha-acid content and essential oils [13,32].

Regarding the production of fresh cones per plant and estimated productivity, Mapuche was the most productive in the 2021 season, differing significantly from other cultivars (Table 5). No differences in production per plant were observed among the Hallertau Mittelfrüher, Northern Brewer, Spalter, and Yakima Gold cultivars. Furthermore, the estimated productivity of Hallertau Mittelfrüher and Northern Brewer was similar to that of Spalter and Yakima Gold; however, Spalter was less productive than Mapuche and more productive than Yakima Gold.

Previous studies have reported that the expected productivity differs between hop cultivars: 2300–2400 kg ha^−1^ for Hallertau Mittelfrüher [33], 1800 kg.ha^−1^ for Mapuche [34], 1600–1800 kg.ha^−1^ for Northern Brewer [35], 1750–2000 kg.ha^−1^ for Spalter [35] and 1800–2000 kg.ha^−1^ for Yakima Gold [36]. 

As with the number of cones, the production and productivity of cones are influenced by vegetative development of the plant and the flowering stage [8,15,28,37]. Consequently, Mapuche, which reached the height of the trellis and flowered in the shortest time, had the highest yield potential. Characteristics such as the number and length of side branches, number of cones per plant, and size and mass of cones, which can also be influenced by vegetative development, directly affect cone yield [28,38].

#### 2.3.2. Season of 2022

In the 2022 harvest, statistically significant differences were observed among hop cultivars in terms of the number of the cones per side branch, number of cones per plant, and cone mass (Table 6). Mapuche and Spalter had the highest mean number of cones per side branch and number of cones per plant, respectively, whereas Hallertau Mittelfrüher and Northern Brewer had the lowest. Yakima Gold had the highest mean cone mass, similar to that of Hallertau Mittelfrüher and Northern Brewer.

In both the second and first seasons, it was observed that the cultivars that stood out in terms of vegetative development, such as Mapuche, Spalter, and Yakima Gold, also had the highest number of cones, either per side branch or per plant. In contrast, Hallertau Mittelfrüher and Northern Brewer, which showed impaired vegetative development, also had the lowest number of cones. Additionally, it was observed that Hallertau Mittelfrüher and Northern Brewer, the cultivars that had a smaller number of cones per lateral branch and per plant, had a higher cone mass. The greater mass of cones in these plants may be due to the lower number of cones per plant and lower source and drain ratios, resulting in greater availability of photoassimilates for the individual development of each cone [31,39]. Significant differences in the physical characteristics of the cones at the 2022 harvest were observed among the hop cultivars, with Yakima Gold and Northern Brewer having the highest average cone lengths (Table 7). Yakima Gold had the highest mean cone width, whereas Hallertau Mittelfrüher had the lowest mean cone width.

According to the cone size classification [40], which considers the length and width of cones, all assessed cultivars had small cones (2.5 cm long and 1.9 cm wide) during the first two evaluated seasons. However, in the first years of hop cultivation, cones are not uniform in terms of size and mass; therefore, it is common for cones to be smaller than expected at this stage [41].

Regarding the production of fresh cones per plant and the estimated productivity in the 2022 season, Mapuche, Spalter, and Yakima Gold reached the highest averages, while Hallertau Mittelfrüher and Northern Brewer had the lowest. For these variables (Table 8), considering the expected productivity of the cultivars, it was observed that for the second season, Yakima Gold, Mapuche, and Spalter had higher productivity than expected, while Hallertau Mittelfrüher and Northern Brewer had lower productivity than expected.

Similar to the first season of 2021, the cultivars that flowered faster after reaching the top of the trellis had the highest yield potential. However, for the second season in 2022, a sharp increase in productivity was expected for all cultivars, as observed in Yakima Gold, as plant production tends to increase due to the stabilization and formation of the nutritional reserves of the rhizomes during the first three years of cultivation [42,43]. Thus, the absence of this high yield may be associated with climatic conditions during plant growth [42].

As previously described, in the 2022 season, immediately after winter pruning, the high volume of precipitation (924 mm between July and October), and consequently a lower incidence of sunlight and the extension of the period of low temperatures (approximately 20 °C), resulted in uneven plant vegetative development [44], necessitating a second cutback of the plants in October, resulting in a lower supply of nutritional reserves for initial plant development [7,26]. Furthermore, in December 2022, when artificial lighting was suspended, the daily photoperiod was the longest of the year (approximately 13.5 h), that is, 1.5 h more than the photoperiod in October when artificial lighting was suspended in the 2021 season. Thus, flowering induction may have been influenced during this season, possibly altering the productive potential of the plants [17].

### 2.4. Chemical Components of Cones

#### 2.4.1. Season of 2021

Differences in the main chemical components of the cones were observed among the cultivars during the 2021 season (Table 9). In this season, the alpha- and beta-acid contents of Yakima Gold were higher than those of Northern Brewer and similar to those of Hallertau Mittelfrüher, Mapuche, and Spalter. Mapuche showed the highest mean concentration of essential oils, while Spalter and Northern Brewer showed the lowest.

Previous studies have reported that the alpha- and beta-acid contents expected for the cultivars are 3.5–5.5% and 4.5–5.0% for Hallertau Mittelfrüher [33], 5.8–6.4% and 4.1–4.8% for Mapuche [34], 8.0–10.0% and 3.0–5.0% for Northern Brewer [35], 3.0–6.5% and 2.0–5.0% for Spalter [35], and 8.8–10.5% and 4.3–5.0% for Yakima Gold [36], respectively. It was observed that in the first season, the means found for alpha-acids were close to what was expected for all cultivars, except for Northern Brewer, which showed a lower mean. The average beta-acid content of all cultivars was lower than expected.

In most cases, the alpha-acid content in the cultivars was higher than the beta-acid content, as observed in this study. In beer, the characteristic bitterness provided by hops is associated with the isomerization of alpha-acids at high temperatures during boiling [13]. Although the influence of beta-acids on the bitterness of beers has yet to be fully elucidated, they are known to be compounds with low solubility in water that do not isomerize during boiling, with approximately 85% of their content being retained in hops after boiling; therefore, only traces of these compounds are found in beers [45]. However, the beta-acid content provides microbiological, chemical, and sensory stability to beers, and the use of hops with a beta-acid content below the expected level, despite minimal interference with the bitterness of beer, may favor contamination during beverage production [13], which can be reversed by the addition of a greater quantity of hops in the process.

The expected concentration of essential oils in hop cones for the studied cultivars is 0.6–1.2 mL.100 g^−1^ for Hallertau Mittelfrüher [35], 1.1 mL.100 g^−1^ for Mapuche [34], 1.5–2.0 mL.100 g^−1^ for Northern Brewer [35], 0.5–1.2 mL.100 g^−1^ for Spalter [35], and 1.9–2.3 mL.100 g^−1^ for Yakima Gold [36]. In the first season, it was observed that the essential oil concentrations of all cultivars except Northern Brewer and Yakima Gold were similar to the expected levels.

In this study, cultivars with essential oil concentrations close to the expected values were considered for their contribution to beer aromas [34,35]. Unlike bitter hops, this type of hop must be added at the end of boiling to avoid the loss of volatile substances present in essential oils due to heat and to give the beer a hoppy aroma. Notably, aroma notes such as floral, spicy, herbaceous, woody, and fruity are synergistically influenced by an extremely complex composition, which can contain up to 1000 compounds of various chemical classes [13,46,47].

The chemical composition of hop cones responds to complex interactions between the genotype and the environment [48]. Each cultivar has the genetic potential to synthesize certain compounds such as soft resins and essential oils [49]. For example, the evaluated cultivars have different aptitudes, with Northern Brewer and Yakima Gold used for two purposes, as they have bittering and aromatic properties, and Hallertau Mittelfrüher, Mapuche, and Spalter being considered aromatic due to their essential oil contents [34,35]. Furthermore, because these compounds are secondary metabolites in plants, the environment plays a role in the regulation of gene expression [48,50]. Consequently, some growing regions are better for some cultivars than for others [51]. This complex interaction can be explained as the environmental conditions of cultivation can regulate gene expression, promoting the development of several phenotypic traits, resulting in phenotypic variability [28,52], which may explain the low quality of Northern Brewer in this cultivation region. This interaction originates from a characteristic known as terroir, which occurs when the environment of a certain growth zone inevitably affects the flavor and aroma properties of the final products. The factors that influence this characteristic are climatic conditions, distinct soil structures, latitude, and associated agricultural practices [53].

#### 2.4.2. Season of 2022

Regarding the chemical components of the cones of cultivars in the 2022 season, Yakima Gold had the highest means in terms of alpha- and beta-acid content and essential oil concentrations, being similar to Spalter in terms of the latter (Table 10). Yakima Gold had the highest mean alpha- and beta-acid contents, followed by Mapuche, while Hallertau Mittelfrüher had the lowest.

In the 2022 season, considering the expected chemical composition for the hop cultivars, it was found that only the alpha-acid content of Mapuche and Spalter and beta-acid content of Spalter and Yakima Gold were close to the expected level. 

The higher levels of beta-acid observed for cultivars in the second season, except for Hallertau Mittelfrüher, may have also been influenced by climatic conditions during the growing season, especially the higher rainfall compared to the first season. Beta-acid accumulations is greater in regions with higher monthly precipitation, which is associated with water availability and accumulation of bitter acids [14].

However, in general, the chemical quality of some cultivars, such as Mapuche, Spalter, and Hallertau Mittelfrüher reached the required global standard in at least one of the evaluated seasons, suggesting that Brazil has potential to develop quality hops that meet the demand of the national beer industry.

### 2.5. Multivariate Analysis

#### 2.5.1. Season of 2021

**Figure 5:**
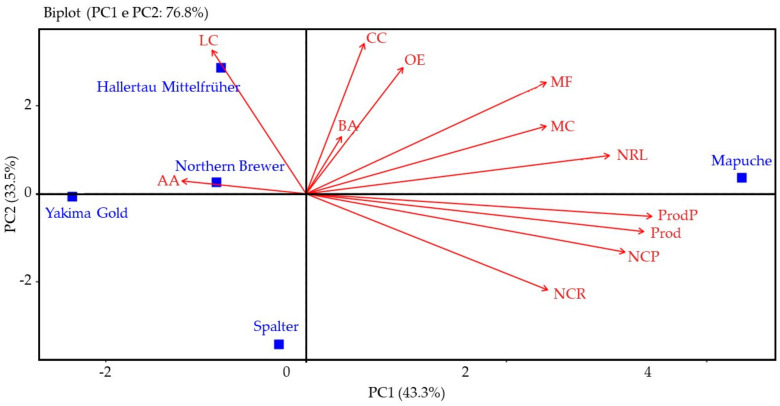
Principal component analysis (APC) for the fresh mass of plants (MF), number of side branches per plant (NRL), number of cones per side branch (NCR), number of cones per plant (NCP), fresh mass of cones (MC), length of cones (CC), width of cones (LC), production of fresh cones per plant (ProdP), estimate of productivity of fresh cones (Prod), alpha-acid content (AA), beta-acid content (BA), and concentration of essential oils (OE) of hop cultivars, season of 2021.

Principal component analysis (APC) indicated that, in the 2021 season, the main components, principal components 1 (PC1) and 2 (PC2), together represented 76.8% of the total variation, with 43.3% of PC1 and 33.5% of PC2 (Figure 5).

**Figure 6:**
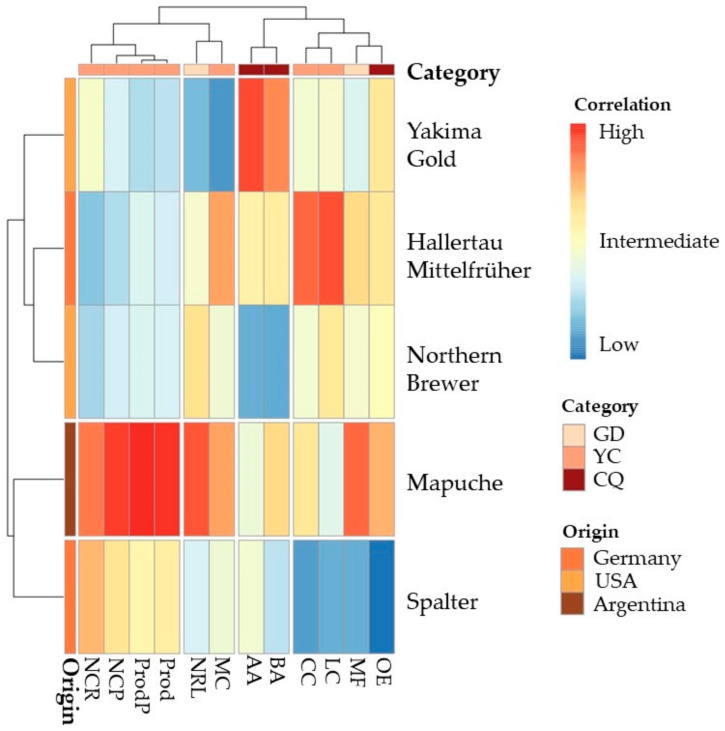
Heatmap using Ward’s hierarchical clustering analysis based on Euclidean distances for fresh mass of plants (MF), number of side branches per plant (NRL), number of cones per side branch (NCR), number of cones per plant (NCP), fresh mass of cones (MC), length of cones (CC), width of cones (LC), production of fresh cones per plant (ProdP), estimate of productivity of fresh cones (Prod), alpha-acid content (AA), beta-acid content (BA), and concentration of essential oils (OE) for hop cultivars, season of 2021. GD = growth development; YC = Yield components; CQ = Chemical quality.

As indicated by PC1, Mapuche had a positive relationship with the fresh mass of plants, number of side branches per plant, number of cones per side branch, number of cones per plant, fresh weight of the cone, length of the cone, production of fresh cones per plant, estimated productivity, beta-acid content, and essential oil concentration in the first season of cultivation. Furthermore, Hallertau Mittelfrüher and Yakima Gold had a positive relationship with cone width and alpha-acid content, and a negative relationship with all other variables. Although Northern Brewer had a positive correlation with alpha-acid content, the heatmap (Figure 6) shows a negative correlation between Northern Brewer and this characteristic, which can be explained by its low contribution (1.25%) to the APC.

PC2 shows that Spalter had a positive relationship with number of cones per side branch, number of cones per plant, production of fresh cones per plant, and estimated productivity, and a negative relationship with the other variables.

As shown in the heatmap (Figure 6), which considered the analysis of correlation and hierarchical grouping, the cultivars formed three and four groups in the first year, respectively. Each cultivar group was correlated with the analyzed variables. The first group consisted of Yakima Gold, Hallertau Mittelfrüher, and Northern Brewer; the second of Mapuche; and the third of Spalter. The first group of variables consisted of the number of cones per side branch, number of cones per plant, production per plant, and estimated productivity; the second group consisted of the number of side branches and fresh mass of the cone; the third group consisted of alpha and beta acid levels; and the fourth group consisted of the width of the cone, length of the cone, fresh weight of the plant, and concentration of essential oils.

The group composed of Hallertau Mittelfrüher, Northern Brewer, and Yakima Gold was negatively correlated with the number of cones per plant, production per plant, and estimated productivity. The group composed of Mapuche was positively correlated with the number of cones per side branch, number of cones per plant, production per plant, and estimated productivity, number of side branches, cone fresh mass, plant fresh mass, and essential oil concentration; however, it had an intermediate relationship with cone length and beta-acid content, and a weak relationship for cone width and alpha-acid content. The group comprising Spalter had an intermediate relationship with the number of cones per side branch, number of cones per plant, production per plant, and estimated productivity, and a negative relationship with the other evaluated components.

#### 2.5.2. Season of 2022

**Figure 7:**
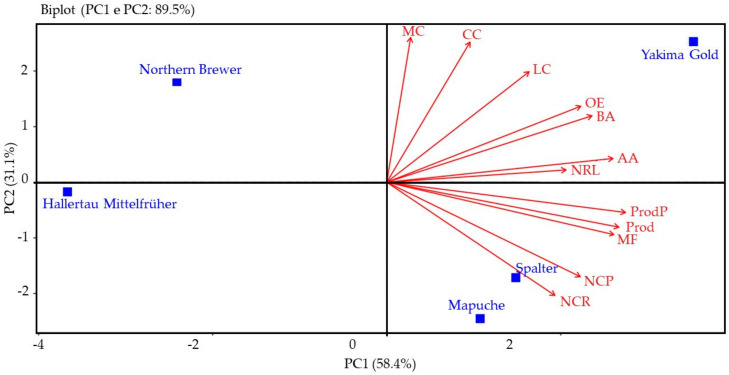
Principal component analysis (APC) of the fresh mass of plants (MF), number of side branches per plant (NRL), number of cones per side branch (NCR), number of cones per plant (NCP), fresh mass of cones (MC), length of cones (CC), width of cones (LC), production of fresh cones per plant (ProdP), estimated productivity of fresh cones (Prod), alpha-acid content (AA), beta-acid content (BA), and concentration of essential oils (OE) of hop cultivars, season of 2022.

Analysis of the main components confirmed that, in the 2022 harvest, PC1 and PC2 together represented 89.5% of the total variation, with 58.4% of PC1 and 31.1% of PC2 (Figure 7).

In this second season of cultivation, PC1, comprising Mapuche, Spalter, and Yakima Gold, had a positive relationship with fresh mass of plants, number of side branches, number of cones per side branch, number of cones per plant, production of fresh cones per plant, estimated yield, alpha- and beta-acid contents, and essential oil concentration, whereas Hallertau Mittelfrüher and Northern Brewer had a negative relationship with these variables.

PC2 showed that Yakima Gold and Northern Brewer had a positive relationship with the variables fresh mass of the cone, cone length, and cone width, while Mapuche, Spalter, and Hallertau Mittelfrüher had a negative relationship.

**Figure 8:**
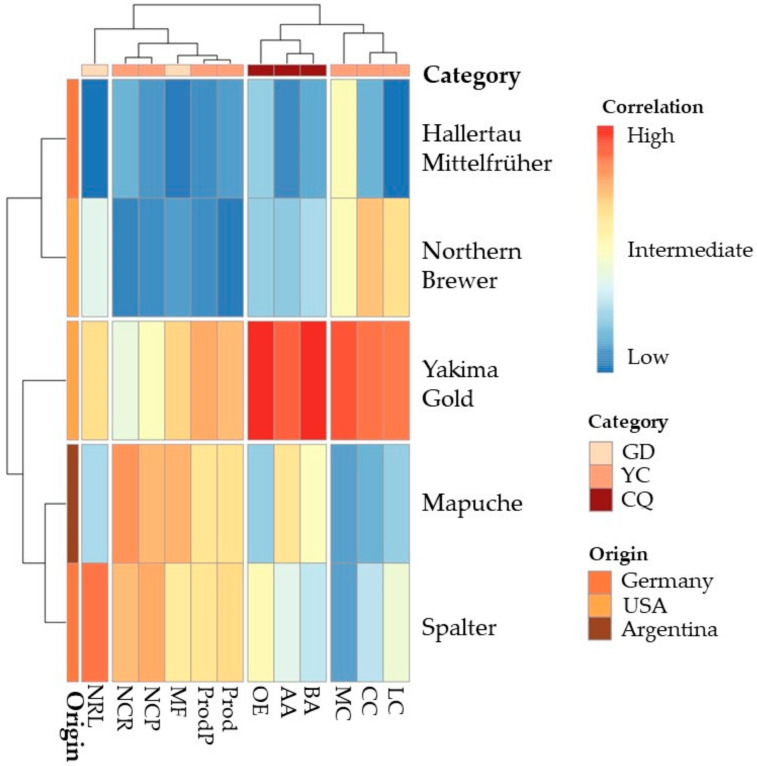
Heatmap created using Ward’s hierarchical clustering analysis based on Euclidean distances for fresh mass of plants (MF), number of side branches per plant (NRL), number of cones per side branch (NCR), number of cones per plant (NCP), fresh mass of cones (MC), length of cones (CC), width of cones (LC), production of fresh cones per plant (ProdP), estimate of productivity of fresh cones (Prod), alpha-acid content (AA), beta-acid content (BA), and concentration of essential oils (OE) for hop cultivars, season of 2022. GD = growth development; YC = Yield components; CQ = Chemical quality.

As shown in the heatmap, in the 2022 season, the cultivars formed three groups and the variables formed four groups (Figure 8). The first group consisted of Hallertau Mittelfrüher and Northern Brewer; the second of Yakima Gold; and the third of Mapuche and Spalter. The first group of variables consisted of the number of side branches per plant; the second of the number of cones per side branch, number of cones per plant, fresh mass of plants, production per plant, and estimated productivity; the third group of the alpha- and beta-acid content and concentrations of essential oils; and the fourth group of fresh cone mass, cone length, and cone width.

The group comprising Hallertau Mittelfrüher and Northern Brewer was negatively correlated with the number of side branches per plant, number of cones per side branch, number of cones per plant, fresh mass of the plants, production per plant, estimated productivity, alpha- and beta-acid content, and concentration of essential oils. The group comprising Yakima Gold showed an intermediate relationship with the number of side branches per plant, number of cones per side branch, number of cones per plant, fresh mass of the plants, production per plant, and estimated productivity, and a positive relationship with the alpha- and beta-acids content, concentration of essential oils, cone mass, cone length, and cone width. The group comprising Mapuche and Spalter had an intermediate relationship with the number of cones per side branch, number of cones per plant, fresh mass of plants, production per plant, and estimated productivity, and a negative relationship with cone mass, cone length, and cone width.

Considering the results of the hop cultivars development in the 2021 and 2022 seasons, it was observed that the Mapuche, Spalter, and Yakima Gold had a promising development performance in terms of productive yield components and chemical components of the cones, demonstrating their cultivation potential for subtropical areas with artificial lighting supplementation.

## 3. Materials and Methods

### 3.1. Experimental Area Description

The trial was conducted in an experimental area of the Federal University of Paraná—UFPR, Setor Palotina, located in the municipality of Palotina, Paraná, Brazil (24°17′40.05″ S; 55°50′23.16″ W, elevation of 332 m a.s.l.). The predominant soil type is red eutroferric Latosol, of basaltic origin and clayey texture, and the climate is Cfa subtropical humid according to the Köppen classification, with an average annual temperature of 20.8 °C, average annual rainfall of 1508 mm [54], and maximum photoperiod in summer of 13.5 h.

The vegetative and productive development of hop (*Humulus lupulus* L.) cultivars Hallertau Mittelfrüher, Mapuche, Northern Brewer, Spalter, and Yakima Gold was assessed during the 2021 and 2022 seasons. The nursery consisted of female plants propagated by cuttings at the State University of Santa Catarina—UDESC, Lajes, SC, Brazil. They were planted in October 2020, trained in a vertical high trellis system (5.5 m high “V” shape), with four bines per plant (two on each supporting wire). Plants in rows were separated by 1.0 m and rows were spaced 3.0 m apart.

During the two seasons assessed, hop bines were cut back close to the ground at the end of the winter season (late July) to stimulate bud development. However, in the 2022 season, the plants had to be cut back close to the ground again in late October because of a high volume of precipitation (accumulated 924 mm between July and October) and the low temperatures observed during this period (approximately 20 °C), which resulted in non-uniform vegetative development of the plants from winter pruning. The monthly averages of air temperature (°C) and rainfall (mm) were determined from daily measurements conducted by the B2K meteorological station located at C-Vale’s headquarters in Palotina, PR, in 2021 (Figure S3) and 2022 (Figure S4).

In both cultivation seasons, similar fertilization, selection, management of branches, removal of leaves at the base of the plant, and weed and pest control regimes were followed. Fertilization was performed via split applications during vegetative development by applying 50 g of NPK 10-10-10 per plant at the beginning of sprouting, leaf development, and side branch formation. After sprouting, the bines in the support wires were selected and trained. Plant leaves were removed during vegetative development to increase vigor, and leaves and old bines were removed at a height of 50 cm from the base portion of the plant. Weeds were mow-controlled in rows and emerald grass was present between rows. Pest control, especially for spotted mites, was conducted by sequential application of citrus essential oil extracts, neem oil, and cupric fungicides.

A double-line drip-irrigation system was installed in the experimental area. As hops are short-day plants, a light supplementation system with LED lamps (spectrum range of 650 nm and 450 nm) with photon flux density of 25 μMol.m^2^.s^−1^ was installed to control flowering during vegetative growth (early season). The lamps were hung at the top of the trellises, and spaced at 10 m so that they hung over the hop plants in the upper part of the trellis. In this phase, the objective was to artificially increase the photoperiod to prevent early flowering, and to ensure that only vegetative development was stimulated. After the plants reached the top of the trellis (height 5.5 m), artificial lighting was permanently turned off so that the short photoperiod stimulated plant flowering.

**Figure 9:**
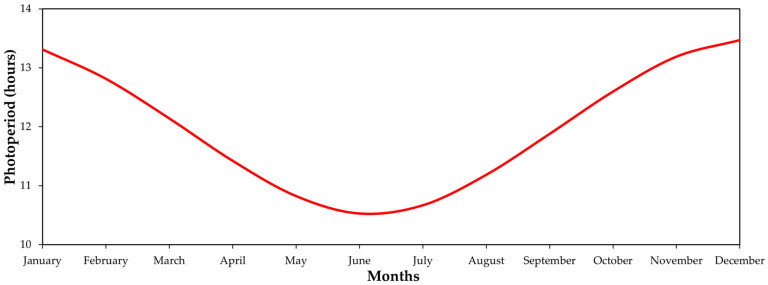
Length of the day in hours (photoperiod) throughout the year in Palotina, PR, Brazil (24° S).

The activation system for these LED lamps was automated and controlled by a timer, and the LEDs were activated for 30 min daily before sunset and kept switched on until 17 h of daily photoperiod was reached. Daily times for automatic switching of light supplementation on and off were determined from the daily photoperiods calculated for the latitude of Palotina, PR (Figure 9), using the equation N = 2/15 arcsin (−tan φ × tan δ), where N = length of day in hours; φ = geographic latitude (negative values for the southern hemisphere); and δ = solar declination, which was calculated using the equation δ = 23.45. sin [360(284 + n)/365], where n is the Julian day. The sine and tangent values were transformed into radians for sine and tangent [55]. Artificial lightning was turned off when the hop plants reached the top of the trellis.

### 3.2. Experimental Design and Assessments

A randomized block design with five treatments (hop cultivars), four replicates, and four plants per plot was used as the statistical model. In both seasons, the hop cultivar phenology, plant growth development, productive yield, and cone chemical components were assessed.

#### 3.2.1. Phenology of Hop Cultivars

**Figure 10:**
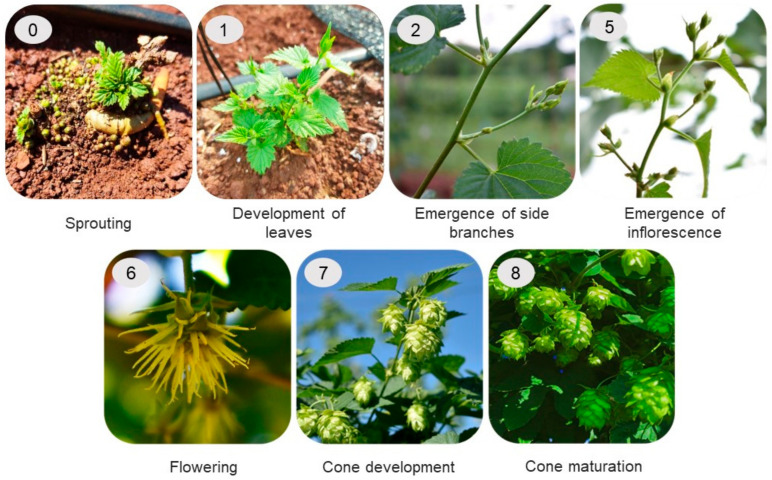
Phenological growth stages of hops (BBCH scale) [56].

The duration of the main phenological stages of the hop cultivars was assessed by evaluating four branches per plot (one branch per plant) every five days, from pruning to harvest. The duration, in days, of the following phenological stages, according to the BBCH scale, was assessed through visual observations: 0. Sprouting, 1. Leaf development, 2. Emergence of side branches, 5. Emergence of inflorescence, 6. Flowering, 7. Cone development, and 8. Cone maturation [56] (Figure 10).

#### 3.2.2. Hop Growth Development

The vegetative development of the hops was assessed using the following variables: plant height, fresh plant mass, and number of side branches per plant. Plant height (m) was defined as the extension from the base of the hop to the insertion of the last leaf emerging from the point of emergence of the first pair of leaves every five days, until the plants reached the height of the trellis (5.5 m).

Plant growth development (m) was analyzed using log-logistic non-linear regressions using the Excel^®^ Solver^®^ tool, with the aim of modeling the data in the form of equations [57]. The following model was used: $Y=\mathrm{Ymax}+(\mathrm{Ymin}\;-\;\mathrm{Ymax})/[1+{\left(\frac{x}{x0}\right)}^{p}]$, where Y is the variable of interest, x the number of accumulated days, and Ymax, Ymin−Ymax, x0, and p are the fitting parameters of the equation, in which Ymax is the highest point obtained, Ymin−Ymax is the difference between the minimum and maximum points, x0 is the number of days that provide 50% of the response of the variable, and p is the slope of the curve.

Fresh plant mass (kg) and number of lateral branches per plant were assessed at harvest.

#### 3.2.3. Yield Components

The harvest point of cones was determined from daily visual sensory observations, and was reached when the cones presented a closed and paper-like appearance texture, with a sudden change from green to yellowish and an intensification of the golden color of lupulin, in addition to a fully developed aroma potential.

The productive yield components assessed at harvest for each cultivar were the number of cones per side branch, number of cones per plant, fresh cone mass, cone length and width, production of fresh cones per plant, and the estimated productivity.

The number of cones per side branch was assessed using the formula (x/y), where x is the number of cones per plant and y is the number of branches per plant. The number of cones per plant was obtained using the formula: $\frac{\left(x\;\times \;100\right)}{y}$, where x = production per plant and y = mass of 100 cones.

The fresh mass of cones (g) was measured using a digital scale. The length and width of the cones were assessed using digital calipers (mm).

The production of fresh cones (kg/plant) for each cultivar was determined by weighing all the cones of each plant on a digital scale. The estimated productivity (kg.ha^−1^) was measured using the formula $x\;\times \;\left(\frac{10,000}{y}\right)$, where x = production of fresh cones per plant and y = area occupied per plant (3 m^2^).

#### 3.2.4. Chemical Components of Cones

To perform chemical analyses of the cones of the hop cultivars, samples of cones (50 g) from each plot were subjected to a cold forced air-drying process (20 °C) until they reached a humidity of 10%. Subsequently, the samples were stored under a vacuum for further evaluation. The chemical components analyzed were alpha- and beta-acid content and essential oil concentration.

Alpha- and beta-acid contents were determined by extracting acids from hop samples, followed by spectrophotometric analysis at three wavelengths [58]. Extraction of the acids was performed using a sample of 2.5 g of hops (ground into a fine powder), which was added to 50.0 mL of methanol. The mixture was stirred for 30 min at room temperature (25 °C), and then allowed to rest for 10 min. Subsequently, filtration using a Millipore membrane filter (0.45 μm) was performed to remove particulate matter. A 50 μL aliquot of the filtrate was placed in a 25 mL volumetric flask and the volume was completed with methanolic NaOH extraction solution (0.5 mL 6M NaOH in 250 mL of methanol). The resulting solution was placed in a quartz cuvette with a 1 cm optical path for evaluation of the visible UV spectrum, using 50 μL of methanol in 25 mL of methanolic NaOH as a blank. The absorbance values were obtained at 275, 325, and 355 nm. From these readings, the following variables were determined: alpha-acid content (%) = $\left[\left(-51.26\;\times \;A355\;\mathrm{nm}\right)+\left(73.79\;\times \;A325\;\mathrm{nm}\right)\;-\;\left(19.07\;\times \;A275\;\mathrm{nm}\right)\right]$ and beta-acid content (%) = $\left[\left(55.27\;\times \;A355\;\mathrm{nm}\right)\;-\;\left(47.59\;\times \;A325\;\mathrm{nm}\right)+\left(5.1\;\times \;A275\;\mathrm{nm}\right)\right]$, where A = absorbance reading at each wavelength.

The essential oil concentration was determined by extracting the essential oils using the water vapor distillation method in a closed loop extractor with an extraction period of 4 h [59]. From each plot, 30 g of dry sample was weighed, ground, and placed in a volumetric flask containing 500 mL of distilled water. The flask was then attached to an extractor and transferred to a heating blanket. The yield of essential oil extracted from plant biomass was calculated using the moisture-free base method using the following formula: essential oil concentration (mL.100 g^−1^) = $(\frac{\mathrm{Vo}}{\mathrm{Bm}-\frac{\mathrm{Bm}\;\times \;U}{100}})\;\times \;100$, where Vo is the volume of oil extracted (mL), Bm is the plant biomass (g), and U is the biomass humidity.

### 3.3. Statistical Analyses

The dataset was subjected to analysis of variance and the F test, and when significant, the means were compared using the Tukey test at 5% probability using R software. The mean number of cones per side branch and the cone fresh mass in the 2021 season were transformed by Box and Cox, and the alpha-acid content was transformed into √x.

Additionally, the mean fresh mass of the plants, number of side branches per plant, number of cones per side branch, number of cones per plant, fresh mass of the cone, cone length and width, production of fresh cones per plant, estimated productivity, alpha-acid content, beta-acid content, and essential oil concentration were subjected to APC, Pearson’s correlation analysis (r), and hierarchical clustering.

For Pearson’s correlation analysis (r) and hierarchical grouping, the variables were classified into the categories growth development (GD), yield components (YC), and chemical quality (CQ), while the cultivars were classified according to the origin of cultivars, i.e., from Germany, United States (USA), and Argentina.

## 4. Conclusions

Among hops grown in subtropical areas with artificial lighting, the cultivar Mapuche, in both seasons, and the cultivars Spalter and Yakima Gold in the second season, were considered early cultivars. In both seasons, the productive yield components were considered positively responsive to the precocity of the cultivars, in which Mapuche in the 2021 season and Mapuche, Spalter, and Yakima Gold in the 2022 season had the highest mean of the number of cones per side branch and per plant, production per plant, and productivity. The chemical composition of the cones of the cultivar Yakima Gold had a positive relationship with alpha- and beta-acid content and with the concentration of essential oils in both seasons.

## Figures and Tables

**Table 1:** Plant fresh mass and number of side arms per plant of hop cultivars during the 2021 season.

Cultivars	Plant Fresh Mass (kg)	N° of Side Branches per Plant
Hallertau Mittelfrüher	1.7	71.9 b
Mapuche	1.9	84.3 a
Northern Brewer	1.5	83.5 a
Spalter	1.2	56.8 c
Yakima Gold	1.4	64.7 bc
CV%	35.8	6.3
F	1.1 ^ns^	27.7 **

Means followed by the same letter in each column do not differ according to Tukey’s test (*p* < 0.05). ^ns^: not significant. **: significant (*p* < 0.01).

**Table 2:** Plant fresh mass and number of side branches per plant of hop cultivars, season of 2022.

Cultivars	Fresh Mass of Plants (kg)	N° of Side Branches per Plant
Hallertau Mittelfrüher	1.3 b	91.7 d
Mapuche	2.3 a	104.8 cd
Northern Brewer	1.4 b	111.5 bc
Spalter	2.1 a	138.1 a
Yakima Gold	2.2 a	126.6 ab
CV%	17.7	7.0
F	8.7 **	20.4 **

Means followed by the same letter in each column do not differ significantly according to Tukey’s test (*p* < 0.05). **: significant (*p* < 0.01).

**Table 3:** Number of cones per side branch, number of cones per plant, and mass of cones of hop cultivars, season of 2021.

Cultivars	N° of Cones per Side Branch	N° of Cones per Plant	Mass of Cones (g)
Hallertau Mittelfrüher	5.9 c	422.8 c	0.4 a
Mapuche	24.0 a	2008.5 a	0.4 a
Northern Brewer	8.2 c	599.2 c	0.3 ab
Spalter	17.9 b	1376.1 b	0.3 ab
Yakima Gold	5.6 c	513.2 c	0.2 b
CV%	12.3	22.0	22.4
F	117.1 **	44.1 **	6.0 **

Means followed by the same letter in each column do not differ according to Tukey’s test (*p* < 0.05). **: significant (*p* < 0.01).

**Table 4:** Length and width of cones of hop cultivars, season of 2021.

Cultivars	Cone Length (cm)	Cone Width (cm)
Hallertau Mittelfrüher	2.5	1.9 a
Mapuche	2.2	1.2 ab
Northern Brewer	2.0	1.5 ab
Spalter	1.6	0.9 b
Yakima Gold	2.0	1.3 ab
CV%	23.2	28.8
F	1.9 ^ns^	3.9 *

Means followed by the same letter in each column do not differ significantly according to Tukey’s test (*p* < 0.05). ^ns^: not significant. * Significant difference (*p* < 0.05).

**Table 5:** Production of fresh cones per plant and yield of hop cultivars, season of 2021.

Cultivars	Production of Fresh Cones (kg/Plant)	Yield of Fresh Cones (kg ha^−1^)
Hallertau Mittelfrüher	0.2 b	579.2 bc
Mapuche	0.9 a	2861.5 a
Northern Brewer	0.2 b	639.6 bc
Spalter	0.4 b	1480.9 b
Yakima Gold	0.1 b	444.8 c
CV%	37.3	38.0
F	19.4 **	19.7 **

Means followed by the same letter in each column do not differ according to Tukey’s test (*p* < 0.05). **: significant (*p* < 0.01).

**Table 6:** Number of cones per side branch, number of cones per plant, and mass of cones of hop cultivars, season of 2022.

Cultivars	N° Of Cones per Side Branch	N° of Cones per Plant	Mass of Cones (g)
Hallertau Mittelfrüher	7.3 cd	624.6 c	0.4 ab
Mapuche	20.1 a	2120.4 ab	0.3 b
Northern Brewer	5.4 d	571.2 c	0.4 ab
Spalter	18.6 ab	2192.5 a	0.3 b
Yakima Gold	12.5 bc	1557.4 b	0.5 a
CV%	23.8	19.1	14.5
F	18.4 **	33.8 **	6.1 **

Means followed by the same letter in each column do not differ according to Tukey’s test (*p* < 0.05). **: significant (*p* < 0.01).

**Table 7:** Length and width of cones of hop cultivars, season of 2022.

Cultivars	Length of Cones (cm)	Width of Cones (cm)
Hallertau Mittelfrüher	1.8 b	1.2 b
Mapuche	1.8 b	1.3 ab
Northern Brewer	2.2 a	1.5 ab
Spalter	1.9 b	1.4 ab
Yakima Gold	2.3 a	1.6 a
CV%	5.1	11.7
F	21.3 **	4.7 *

Means followed by the same letter in each column do not differ according to Tukey’s test (*p* < 0.05). *: significant (*p* < 0.05). ** Significant difference (*p* < 0.01).

**Table 8:** Production of fresh cones per plant and yield of hop cultivars, season of 2022.

Cultivars	Production of Fresh Cones (kg/Plant)	Yield of Fresh Cones (kg ha^−1^)
Hallertau Mittelfrüher	0.2 b	811.1 b
Mapuche	0.6 a	2051.0 a
Northern Brewer	0.2 b	657.6 b
Spalter	0.6 a	2122.9 a
Yakima Gold	0.7 a	2251.0 a
CV%	19.1	19.1
F	26.5 **	26.5 **

Means followed by the same letter in each column do not differ according to Tukey’s test (*p* < 0.05). **: significant (*p* < 0.01).

**Table 9:** Alpha- and beta-acid contents and essential oil concentrations of hop cone cultivars, season of 2021.

Cultivars	Alpha-Acid (%)	Beta-Acid (%)	Essential Oils (mL.100 g^−1^)
Hallertau Mittelfrüher	5.9 ab	1.8 ab	1.1 ab
Mapuche	5.0 ab	1.9 ab	1.2 a
Northern Brewer	3.0 b	1.2 b	1.0 b
Spalter	5.1 ab	1.4 ab	0.6 c
Yakima Gold	8.5 a	2.1 a	1.1 ab
CV%	16.5	17.9	7.2
F	5.1 *	5.5 **	34.8 **

Means followed by the same letter in each column do not differ according to Tukey’s test (*p* < 0.05). **: significant (*p* < 0.01). * Significant difference (*p* < 0.05).

**Table 10:** Alpha- and beta-acid contents and essential oil concentrations of hop cone cultivars, season of 2022.

Cultivars	Alpha-Acid (%)	Beta-Acid (%)	Essential Oils (mL.100 g^−1^)
Hallertau Mittelfrüher	2.3 d	1.4 d	0.8 b
Mapuche	5.8 b	2.5 b	0.8 b
Northern Brewer	3.2 cd	1.8 c	0.8 b
Spalter	4.2 c	1.9 c	1.0 ab
Yakima Gold	7.4 a	4.0 a	1.3 a
CV%	10.2	6.3	14.3
F	75.5 **	190.2 **	12.0 **

Means followed by the same letter in each column do not differ according to Tukey’s test (*p* < 0.05). **: significant (*p* < 0.01).

## Data Availability

Data are available upon request.

## Supplementary Materials

The following supporting information can be downloaded at: https://www.mdpi.com/article/10.3390/plants12101971/s1, Figure S1: Growth development of hop plants, season of 2021. A: ‘Hallertau Mittelfrüher’; B: ‘Mapuche’; C: ‘Northern Brewer’; D: ‘Spalter’; E: ‘Yakima Gold’; (-----): estimated plant height (m). Figure S2: Growth development of hop plants, season of 2022. A: ‘Hallertau Mittelfrüher’; B: ‘Mapuche’; C: ‘Northern Brewer’; D: ‘Spalter’; E: ‘Yakima Gold’; (-----): estimated plant height (m). Figure S3: Average air temperature and precipitation in Palotina, PR, Brazil in 2021. Figure S4: Average air temperature and precipitation in Palotina, PR, Brazil in 2022.
