# Supplementary material for: Performance of Hop Cultivars Grown with Artificial Lighting under Subtropical Conditions
Source: Plants (Basel). 2023 May 12;12(10):1971. doi: 10.3390/plants12101971 (PMC10222731; doi:10.3390/plants12101971)
Supplement: Supplementary file 1 [file plants-12-01971-s001.zip › plants-2397096-supplementary.pdf]

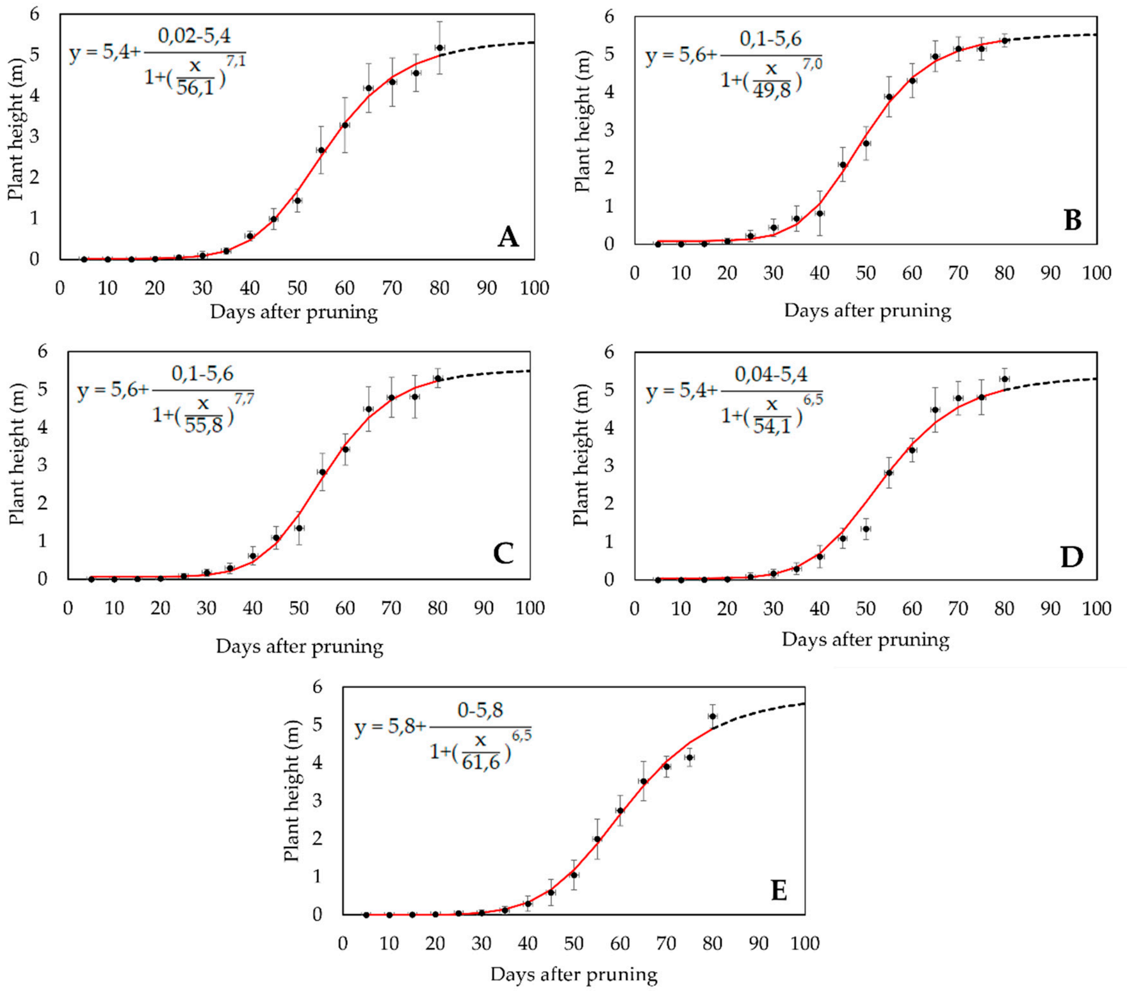

Figure S1. Growth development of hop plants, season of 2021. A: ´Hallertau Mittelfrüher´; B: ´Mapuche´; C: ´Northern Brewer´; D: ´Spalter´; E: ´Yakima Gold´; (-----): estimated plant height (m).

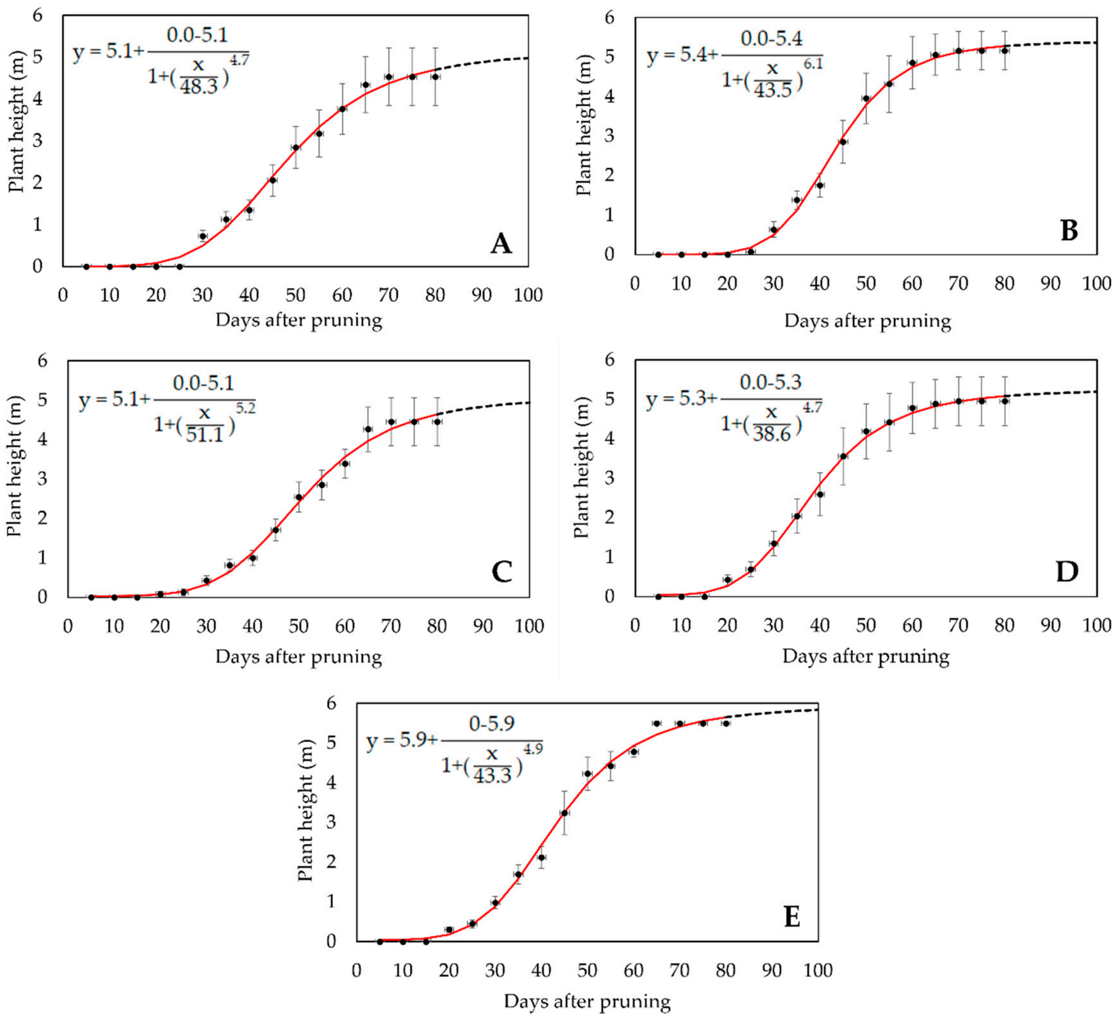

Figure S2. Growth development of hop plants, season of 2022. A: ´Hallertau Mittelfrüher´; B: ´Mapuche´; C: ´Northern Brewer´; D: ´Spalter´; E: ´Yakima Gold´; (-----): estimated plant height (m).

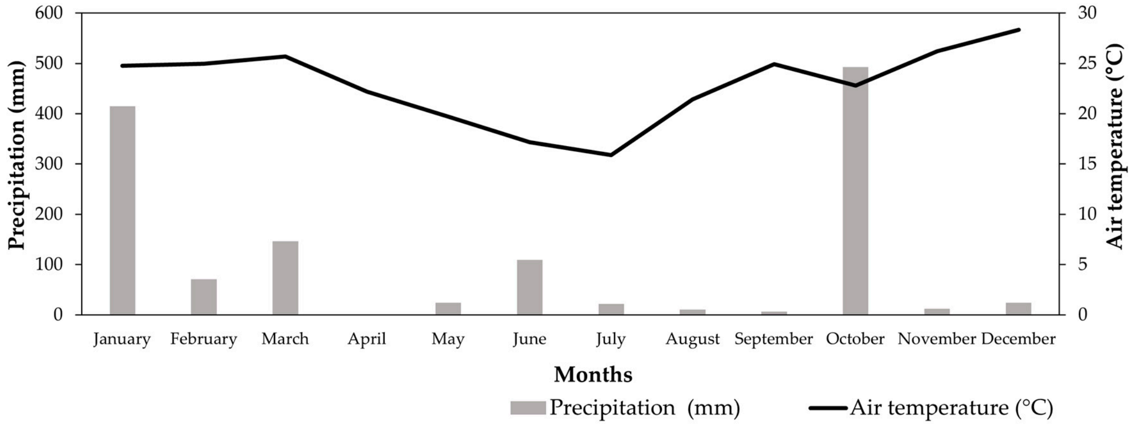

Figure S3. Average air temperature and precipitation in Palotina, PR, Brazil in 2021.

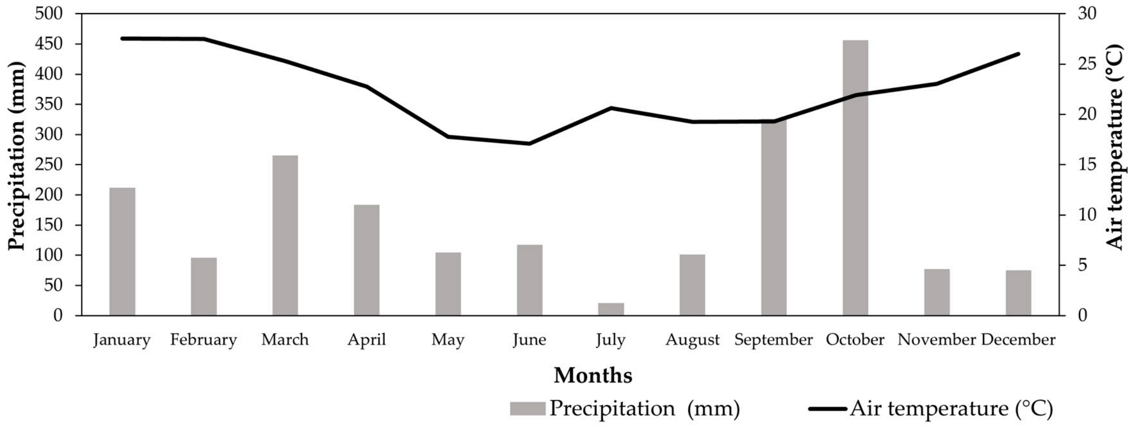

Figura S4. Average air temperature and precipitation in Palotina, PR, Brazil in 2022.
